# In Vitro Effects of *Rumex confertus* Extracts on Cell Viability and Molecular Pathways in MCF-7 Breast Cancer Cells

**DOI:** 10.3390/antiox14070879

**Published:** 2025-07-18

**Authors:** Levent Gülüm, Emrah Güler, Fatma Lale Aktaş, Ayşe Büşranur Çelik, Hilal Yılmaz, Yusuf Tutar

**Affiliations:** 1 Department of Plant and Animal Production, Mudurnu Süreyya Astarcı Vocational College, Bolu Abant İzzet Baysal University, Bolu 14030, Türkiye; 2 Innovative Food Technologies Development Application and Research Center, Bolu Abant İzzet Baysal University, Bolu 14030, Türkiye; 3 Department of Horticulture, Faculty of Agriculture, Bolu Abant İzzet Baysal University, Bolu 14030, Türkiye; emrahguler6@gmail.com; 4 Department of Biochemistry, Hamidiye Faculty of Pharmacy, University of Health Sciences, Istanbul 34668, Türkiye; lale.aktas123@gmail.com; 5 Molecular Biology and Genetics, University of Health Sciences, Istanbul 34668, Türkiye; ayseclk1899@gmail.com; 6 Plant and Animal Production Program, Izmit Vocational School, Kocaeli University, Kocaeli 41285, Türkiye; hilal.yilmaz@kocaeli.edu.tr; 7 Department of Basic Pharmaceutical Sciences, Division of Biochemistry, Faculty of Pharmacy, University of Health Sciences, Istanbul 34668, Türkiye; 8 Faculty of Medicine, Division of Biochemistry, Recep Tayyip Erdogan University, Rize 53100, Türkiye

**Keywords:** *Rumex confertus*, anticancer activity, breast cancer, MCF-7 cell line, signaling pathways

## Abstract

*Rumex confertus* (RC), a plant known for its traditional medicinal uses, has shown potential anticancer properties, particularly due to its rich phenolic content. Despite its promising bioactivity, its effects on breast cancer cells remain underexplored. Here, we investigated the cytotoxic effects of RC extracts on MCF-7 breast cancer cells, employing various solvents for extraction. This study revealed that the hexane extract significantly reduced the cell viability, with an IC_50_ of 9.40 µg/mL after 96 h. The gene expression analysis indicated a substantial modulation of transcriptional networks, including the upregulation of pluripotency-related genes and the downregulation of differentiation markers. The findings suggest that the RC extract may induce a shift towards a less differentiated, stem-like state in cancer cells, potentially enhancing malignancy resistance. This study underscores the potential of RC as a candidate for breast cancer treatment, and a further investigation into its therapeutic applications is suggested.

## 1. Introduction

*Rumex confertus* (RC), commonly known as Asiatic dock, is a plant species from the *Polygonaceae* family that has been utilized in traditional medicine for many years [1]. Owing to its rich phytochemical composition, recent research has increasingly focused on its diverse biological activities, with particular attention given to its antioxidant, anticancer, and antimicrobial properties [2]. The phenolic compounds present in RC have garnered significant scientific interest due to their recognized health benefits [3]. These bioactive components—including flavonoids, hydroxycinnamic acids, tannins, and anthraquinones—are widely noted for their antioxidant, anti-inflammatory, antimicrobial, and anticancer effects [4].

Studies have consistently highlighted the strong antioxidant activity of RC, largely attributed to its phenolic constituents [5]. These phenolic compounds act as powerful antioxidants, effectively neutralizing free radicals and thereby helping to prevent oxidative stress in biological systems [6]. Since oxidative stress is linked to cellular damage and the development of various chronic diseases, including cancer [6,7], the high phenolic content of RC suggests a promising role in mitigating such effects. Notably, compounds such as hydroxycinnamic acids and flavonoids are recognized for their capacity to combat cancer and other chronic conditions [8], further supporting the therapeutic potential of RC.

Recent studies have demonstrated that plant-derived secondary metabolites (SPMs) exert anticancer effects by targeting multiple signaling pathways. Advanced techniques such as Raman-SIS and isotope probing enable the precise monitoring of these compounds’ cellular mechanisms. Metabolites like gallic acid, noscapine, paclitaxel, and curcumin are particularly noteworthy due to their involvement in apoptosis, cell cycle arrest, and metastasis inhibition [9].

The *Rumex* genus comprises around 200 species. Research has focused on only about 50 of these. Its bioactive compounds, including anthraquinones, flavonoids, and polysaccharides, show cytotoxic and antiproliferative effects on cancer cells. Notably, emodin and chrysophanol demonstrate potential as therapeutic agents by inducing apoptosis and modulating the cell cycle, and many compounds remain to be explored [3].

In a study investigating the cell-killing potential of *Rumex abyssinicus* against HeLa cells, the results demonstrated that *R. abyssinicus* is a promising candidate for cancer treatment [10]. The potential cytotoxic effect of *R. abyssinicus* on breast cancer cell lines was investigated. The study found that the chloroform extract exhibited a potential in vitro cytotoxic activity against MCF-7 breast cancer cell lines [11]. Extracts from other *Rumex* species, like *R. crispus*, have demonstrated potent cytotoxic effects against breast cancer cell lines, such as MCF-7 [12,13], suggesting that RC may also exhibit an effective anticancer activity through similar mechanisms. Anthraquinones are recognized for their ability to induce cell death and inhibit tumor growth, which highlights their significance in the treatment of cancer [14].

In addition to its antioxidant activity, the anticancer potential of RC has recently attracted considerable attention. Key components of this plant—such as anthraquinones, flavonoids, and hydroxycinnamic acids—have shown notable promise in cancer therapy through several mechanisms, including the induction of apoptosis (programmed cell death) in cancer cells, the inhibition of cell proliferation, and the suppression of carcinogenic pathways [12,13]. The antibacterial and antifungal effects of RC’s phenolic compounds broaden the therapeutic applications of the plant [12]. Traditionally used for various ailments in folk medicine, RC also stands out as a promising candidate for the development of natural health products in modern pharmacology [15]. The combination of antimicrobial and antioxidant properties enhances its potential utility in both traditional and contemporary medicine [16]. Research on RC to determine the contributions of its phenolic compounds and other bioactive components to health is essential [15]. Studies on the anticancer, antioxidant, and antimicrobial properties of RC could lead to new treatment options in the pharmaceutical and food supplement sectors [17,18]. Previous studies have generally only investigated the cytotoxic effects on specific cell lines. However, this study aimed to determine the most appropriate extraction solvent to detect the cytotoxic effects of RC extracts, to determine their biochemical contents, and to examine the changes in signaling pathways with 46 genes at the molecular level in cancerous breast cells. While current studies on RC provide valuable insights, there are no in vitro or in vivo studies that thoroughly examine the plant’s biological activity on breast cancer cells. In this regard, this study evaluated the cytotoxic effects of the RC extract on the MCF-7 breast cell line and assessed its effects through genomic induction.

## 2. Materials and Methods

### 2.1. Plant Sampling and Preparation

RC samples were obtained from the natural products market in Makhachkala, Dagestan. The dried and powdered samples were dissolved in 25 mL of solvent (ethyl acetate, hexane, or methanol) using 2 g of the RC powder. This mixture was left at room temperature for 72 h. Following the incubation period, the mixture was sonicated for 15 min using an ultrasound device from HY Technologies (Cairo, Egypt). The solution was then filtered through Whatman No. 1 filter paper, and the sonication process was repeated with an equal volume of fresh solvent to ensure maximum extraction efficiency. After sonication, the combined extracts were centrifuged at 10,000 rpm for 10 min. These extracts were analyzed to determine their total content of phenolics, anthocyanins, proteins, carbohydrates, and flavonoids, as well as to evaluate their antioxidant properties.

For cytotoxicity studies, the resulting supernatants were concentrated under vacuum using a rotary evaporator, and all crude fractions were stored at −20 °C. The extracts were then dissolved in dimethyl sulfoxide (DMSO) to prepare 1 mg/mL stock solutions. Required concentrations for the experiments were subsequently prepared from this stock solution [19,20].

### 2.2. Biochemical Assays

The chemical composition of RC was analyzed using techniques such as UV Spectrometry. Active compounds such as flavonoids, alkaloids, total phenolics, total antioxidants, total oxidation proteins, total antioxidants, and total anthocyanins were identified and quantified.

#### 2.2.1. Total Phenolic Content (TPC)

The TPC was determined by a modified method proposed by Waterhouse [21]. The method was modified to fit a microplate reader as follows. Briefly, 50 microliters of sample was mixed with 100 microliters of distilled water and 5 microliters of Folin–Ciocalteu reagent and vortexed. After, 30 microliters of 7% Na_2_CO_3_ solution was added and vortexed again. The standards that were prepared by serial dilution of 0.2 mM gallic acid went through the same procedure. The samples and standards were incubated in the dark under ambient conditions for 2 h, and readings were performed using a microplate reader (Thermo Scientific™ Multiskan™, Waltham, MA, USA) in 760 nm wavelength. The results were expressed as mM GAE (gallic acid equivalents).

#### 2.2.2. Total Flavonoid Content (TFC)

Total flavonoid content in extracts and fractions was determined using a modified method from [22]. Briefly, 100 μL of plant extract or standard solution was mixed with 300 μL of 5% sodium nitrite and incubated for 5 min. Then, 300 μL of 10% aluminum chloride (AlCl_3_) was added, followed by a 6 min incubation at 27 °C. After adding 2 mL of 0.5 M sodium hydroxide (NaOH), the volume was adjusted to 10 mL with distilled water. Absorbance was measured at 430 nm using a spectrophotometer (DLAB, SP1000, Beijing, China). Quercetin, diluted from a 2 mM solution, served as the standard for a calibration curve (r^2^ = 0.9996), with results expressed in millimoles (mM).

#### 2.2.3. Total Anthocyanin Content

The total anthocyanin content was measured using a 30% ethanol solution in an acidic medium. Specifically, 1500 microliters of a mixture consisting of 70% pure water, 30% ethanol, and 1% hydrochloric acid (HCl) was added to 500 microliters of the sample. This mixture was vortexed and then allowed to sit at room temperature for 15 min. Afterward, the measurement was taken at a wavelength of 540 nm. The results were calculated as mg/L of Malvidin-3-glucoside equivalent using the formula:

Tant = 16.7 × A540 × Df

In this formula, Df represents the dilution factor [23].

#### 2.2.4. Total Soluble Protein

Total soluble protein content was used by modifying the method reported by [24]. Briefly, 50 microliters of samples or bovine serum albumin (BSA) standards were mixed with 0.2 mL of Bradford reagent and incubated for 15 min at ambient conditions. After the incubation, samples and standards were read at 595 nm wavelength using a microplate reader (Thermo Scientific™ Multiskan™, Waltham, MA, USA). Results were expressed as %.

#### 2.2.5. Total Soluble Carbohydrates

The phenol–sulfuric acid method was applied with slight modifications to measure total soluble carbohydrate content [25]. In brief, 10 microliters of phenol diluted with ethanol and 180 microliters of concentrated sulfuric acid were added to 10 microliters of the sample. A 20% glucose standard solution was prepared through serial dilution following the same procedure. Results were expressed as a percentage based on the calibration curve obtained from glucose.

#### 2.2.6. Antioxidant Activity Determination

##### DPPH Radical Scavenging Activity

To determine the DPPH (2,2-diphenyl-1-picrylhydrazyl) scavenging activity, we adapted the method described by [26], with minor modifications. In brief, DPPH was dissolved in ethanol to achieve an absorbance between 700 and 800 at 517 nm. Next, 190 microliters of the DPPH solution was mixed with 10 microliters of the samples, and the absorbance was measured using a microplate reader (Thermo Scientific™ Multiskan™, Waltham, MA, USA) after a 15 min incubation. Ascorbic acid, diluted from a 2 mM solution, was used as a standard following the same procedure. The resulting curve allowed for the calculation of DPPH scavenging values in the samples, expressed in mM.

The concentration of the extract or standard that achieved a 50% reduction in the initial DPPH^•^ concentration was defined as EC50, calculated from the percentage of free radical scavenging activity against the tested concentrations, reported as EC50 = mg/mL.

##### Copper (II) Ion Reducing Antioxidant Capacity (CUPRAC)

The copper (II) ion reducing antioxidant capacity of the extracts was assessed using a modified method based on [27]. A reagent was prepared by combining 10 mM copper (II) nitrate solution, 7.5 mM neocuproine (Nc) solution, and 1.0 M ammonium acetate (NH_4_Ac). In a microplate, 190 microliters of this reagent was added to 10 microliters of the sample. The mixture was vortexed and allowed to stand at room temperature for 20 min. Ascorbic acid was used as a standard, serially diluted from a 2 mM concentration, and treated in the same manner. The absorbance of the samples and standards was measured at 450 nm using a microplate reader (Thermo Scientific™ Multiskan™, Waltham, MA, USA), with results reported in millimolar (mM).

##### ABTS Free Radical Scavenging Activity

The ABTS^•+^ radical cation scavenging activity was measured with minor modifications to the method established by [28]. A 7 mM ABTS^•+^ solution, containing 2.45 mmol of potassium persulfate, was prepared and left in the dark for 12–16 h to promote radical formation at ambient conditions. The resulting dark-blue solution was then diluted with 20 mM sodium acetate (pH 4.5) until its absorbance reached 0.7 ± 0.01 nm at 734 nm.

In a microplate, 190 microliters of the ABTS solution was mixed with 10 microliters of the sample or standard, with standards prepared by serially diluting a 2 mM ascorbic acid solution. The mixture was incubated for 15 min at room temperature. Results were calculated using the equation derived from the ascorbic acid standards and expressed in millimolar (mM).

##### Ferric (III) Reducing Antioxidant Power (FRAP)

This study followed the standard procedure outlined by [29]. The reagent was diluted 1:1 for analysis. In a microplate, 10 microliters of both the sample and standard solution were combined with 190 microliters of the FRAP reagent. A 0.2 mM solution of L-ascorbic acid was used as the standard, prepared through serial dilution. The readings were performed at 593 nm using a microplate reader (Thermo Scientific™ Multiskan™, Waltham, MA, USA). Samples’ antioxidant capacity was calculated using the equation derived from the L-ascorbic acid standards and expressed in millimoles (mM).

### 2.3. Cell Culture and In Vitro Experiments

Human breast cancer cell line MCF-7 was cultured under standard conditions. The cells were treated with various concentrations of RC extract, and cell viability was measured using the MTT assay. Total RNA extraction, purification, and cDNA synthesis analyses were conducted. Additionally, apoptosis induction and cell cycle analyses were assessed using flow cytometry and Annexin V staining.

#### 2.3.1. Cell Culture Conditions and MTT Assay

In this study, MCF-7 breast cancer cells were obtained from ATCC. The cell line was cultured in DMEM-High Glucose (Euroclone, Pero, Italy) medium supplemented with 10% fetal bovine serum (FBS) (Euroclone, Pero, Italy) and 1% penicillin–streptomycin (Euroclone, Pero, Italy) and incubated at 37 °C in a humidified atmosphere containing 5% CO_2_. Once the flasks reached 80–90% confluence, the cells were detached using 0.25% trypsin–EDTA (Euroclone, Pero, Italy).

To investigate the effects of RC extracts on cell viability, MCF-7 cells were first seeded into 96-well plates at a density of 5000 cells per well. After 24 h, the media in the wells were removed, and plant extracts from RC were applied. These concentrations were applied as follows: 200.00, 100.00, 50.00, 25.00, 12.50, 6.25, and 3.125 mg/mL. Solvents at the same concentrations were applied to the control wells. To evaluate the time-dependent effects of the extracts, cells were incubated separately for 48 and 96 h with media containing the extracts. Following this incubation period, cell viability analysis was performed using the MTT (3-(4,5-Dimethylthiazol-2-yl)-2,5-Diphenyl Tetrazolium Bromide) (Sigma-Aldrich, Saint Louis, MO, USA) assay according to the manufacturer’s instructions. Briefly, MTT solution (5 mg/mL) was added to the wells at 20% of the total volume and incubated for 3 h. After this, the media were removed, and the resulting formazan crystals were dissolved in 100 μL of DMSO. Absorbance values were then recorded at a wavelength of 570 nm using a microplate reader (Thermo Scientific, Multiskn Go, Waltham, MA, USA).

#### 2.3.2. IC_50_ Determination

IC_50_ values were determined by nonlinear regression analysis using the “Log(inhibitor) vs. response–variable slope” model implemented in GraphPad Prism version 8.0.2. This method fits the dose–response data to a sigmoidal curve, allowing for the calculation of the inhibitor concentration that reduces cell viability by 50%. The variable slope parameter accounts for differences in the steepness of the dose–response curve, providing a more accurate estimation of IC_50_ values.

#### 2.3.3. Total RNA Extraction, Purification, and cDNA Synthesis with Real-Time qPCR

The effects of RC extracts on signaling pathways were determined by real-time quantitative PCR (RT-qPCR). For Total RNA isolation, 350,000 cells per well were seeded into 6-well plates, and after 24 h of incubation, the compound with the lowest IC50 value was applied at its IC50 concentration. Following 96 h of incubation, RNA isolation was performed using the innuPREP RNA Mini Kit 2.0 (Innuscreen GmbH, Berlin, Germany) according to the manufacturer’s instructions. The RNA content obtained was measured using a spectrophotometer (Eppendorf BioSpectrometer^®^, Hamburg, Germany), with results presented as ng/μL RNA and an A260/280 ratio. cDNA synthesis was carried out using the Wonder RT-cDNA Synthesis Kit (Euroclone, Milan, Italy) according to the manufacturer’s protocol. qPCR amplification was performed using the Thermo Fisher 5020 Arktik Thermal Cycler (Thermo Fisher, USA).

The expression changes of 46 genes related to signaling pathways were analyzed by RT-qPCR using SYBR Green Master Mix (EuroClone, Pero, Italy) on the Analytical Jena qTOWER3 RT-PCR instrument (Analytik Jena, Jena, Germany). The gene set was selected based on a widely used gene panel that is described in the literature. This panel covers pathways related to the cell cycle, apoptosis, stem cell-like characteristics, and metastasis. Signaling pathways GAPDH and ACTB were used as reference genes. Results were analyzed using the Livak method [30].

### 2.4. Metabolic Responses of Cancer Cells

The gene expression values from cells treated with RC-hexane were analyzed using the Reactome database (https://reactome.org). This analysis identified the metabolic pathways that showed significant interactions, and the relevant images from the same site were included. Only interactions with a *p*-value of ≤0.05 were considered in this evaluation. Pathway enrichment analysis was conducted using the, employing the hypergeometric test to identify significantly enriched biological pathways. Reactome Pathway Knowledgebase To address the issue of multiple hypothesis testing and minimize false positives, *p*-values were adjusted using the Benjamini–Hochberg method. A false discovery rate (FDR) threshold of 0.05 was applied, as this is widely accepted in transcriptomic and systems biology analyses, ensuring a balance between sensitivity and statistical reliability.

### 2.5. Flow Cytometer Analysis

In flow cytometry analysis, Beckman Coulter CytoFLEX (Brea, CA, USA) cytometer was used with CytExpert software (ver. 2.4.0.28). At least 10,000 events were analyzed per sample. Debris was eliminated by excluding diseases with low signal on the FSC-A vs. SSC-A plot and by excluding populations that exhibited deviation from the single cell diagonal on the FSC-A vs. FSC-H plot in pairs.

#### 2.5.1. Cell Cycle Analysis

Cell cycle analysis was performed using the Sigma-Aldrich Mak344 Cell Cycle Analysis kit, following the specified protocol. MCF-7 cells were cultured at a concentration of 3 × 10^5^ cells per well, and treatments were applied for 96 h at the determined IC50 values for the samples. At the end of the treatment period, the cells were washed with 500 μL of phosphate-buffered saline (PBS) and then trypsinized. The cell pellet was collected by centrifugation at 400 rpm for 5 min. Following this, the cells were washed with 2 mL of 1X cell cycle assay buffer and centrifuged again at 400 rpm for 5 min. The supernatant was discarded, and the cells were fixed by slowly and carefully adding 2 mL of cold 70% ethanol to the pellets. The ethanol-treated cells were kept on ice for a minimum of 30 min. Afterward, the samples were centrifuged once more, and the supernatant was discarded. The pellets were washed again with 2 mL of 1X cell cycle assay buffer, ensuring that no cells were lost when removing the supernatant. Next, 500 μL of the staining solution was prepared using 1X buffer solution containing 100 μL of enzyme A solution and 400 μL of nuclear dye, which was then added to the cells. These were incubated at room temperature in a dark environment for 30 min before being analyzed using flow cytometry [31].

#### 2.5.2. Apoptosis Analysis

For apoptosis detection, we utilized the FITC Annexin-V Apoptosis Detection Kit with Propidium Iodide (PI), specifically the ApopNexin™ FITC (Merck, Darmstadt, Germany). MCF-7 cells were seeded in 6-well plates at a concentration of 1 × 10^5^ cells per well. After seeding, the cells were cultured with cytotoxic concentrations for 96 h. Following the incubation with the apoptotic cell extracts, the medium was removed, and the cells were washed with PBS. They were then lifted using trypsin–EDTA and centrifuged at 400 rpm for 5 min. The cells were washed twice with 5 mL of pre-cooled phosphate-buffered saline (PBS). Subsequently, the cells were treated with 1 mL of cold 1X Binding Buffer (Sigma-Aldrich, St. Louis, MO, USA). From this mixture, 200 μL was transferred to a flow cytometry tube, while the remaining cells were kept on ice. To the sample tube, 3 μL of ApopNexin™ FITC and 2 μL of 100X propidium iodide (PI) (Sigma-Aldrich, USA) were added, and the mixture was mixed well. The resulting mixture was incubated at room temperature in the dark for 15 min. After the incubation, the samples were analyzed using flow cytometry to obtain the data [32].

### 2.6. Experimental Design and Statistics

The extraction study was conducted using a factorial design with three replications. The obtained values were subjected to two-way analysis of variance (ANOVA) to determine whether the effects of variation sources (pistachio wastes and solvents) were significant. Fisher’s Least Significant Differences (LSDs) test was utilized at the α = 0.05 level to assess the differences across means. The study was designed using JMP Pro16 software (SAS, Cary, NC, USA). Cell viability analyses were performed using GraphPad Prism version 8.0.2.

## 3. Results and Discussion

### 3.1. Biochemical Composition

**Figure 1:**
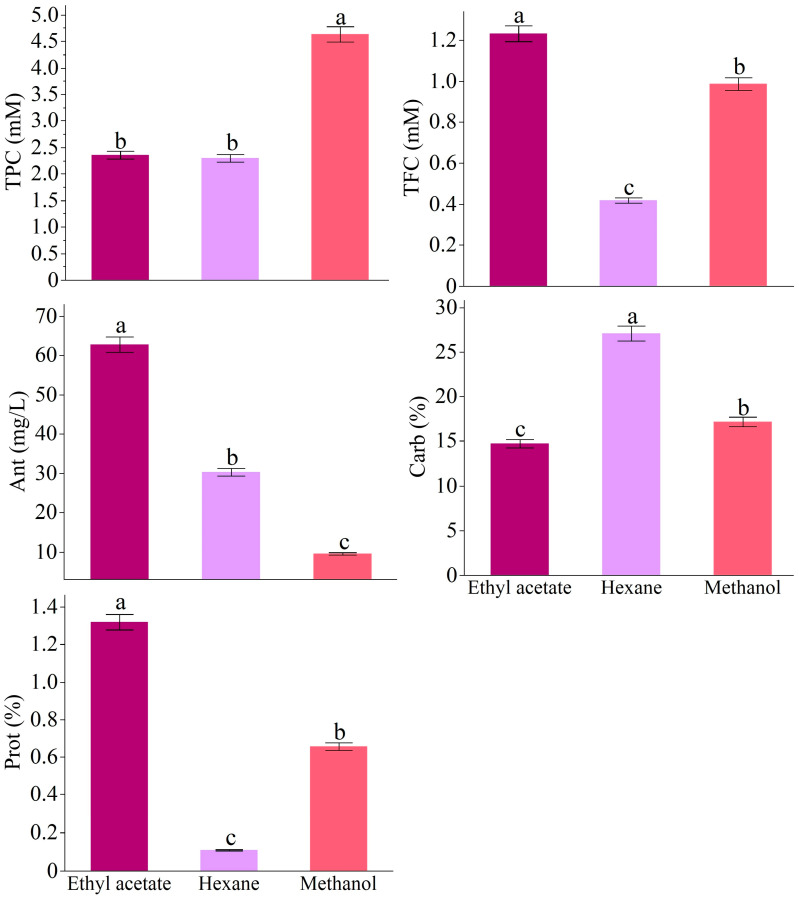
Total phenolic (TPC), total flavonoid (TFC), total anthocyanin (Ant), total protein (Prot), and total carbohydrate (Carb) contents according to the solvents. The data were expressed as mean ± standard deviation. Different letters on the top of the bars indicate a significance at *p* < 0.05 according to Fisher’s LSD test.

The total soluble phenolic content (TPC) showed that the methanol extract had the highest value at 4.63 mM, followed by ethyl acetate at 2.36 mM and hexane at 2.30 mM. The total soluble clavonoid content (TFC) revealed that the highest value was found in the ethyl alcohol extract at 1.23 mM, whereas the hexane extract was the lowest at 0.42 mM. The total amounts of anthocyanins were compared among solvents, showing that the extract obtained with ethanol had the highest total anthocyanin content at 62.80 mg/L, followed by the hexane extract at 30.26 mg/L, while the methanol extract had the lowest amount at 9.53 mg/L. The total soluble carbohydrate content was significantly influenced by the type of solvent used (*p* ≤ 0.001), with the hexane extract having the highest total soluble carbohydrate content at 27.09%, while the methanol extract had the lowest at 14.73%. Additionally, the total soluble protein content in all extracts demonstrated a significant change (*p* ≤ 0.001). Upon the examination of the protein content, it was found that the ethyl alcohol extract had the highest level at 1.32%, followed by the methanol extract at 0.66%. The hexane extract showed the lowest protein content (Figure 1).

### 3.2. Antioxidant Capacities

**Figure 2:**
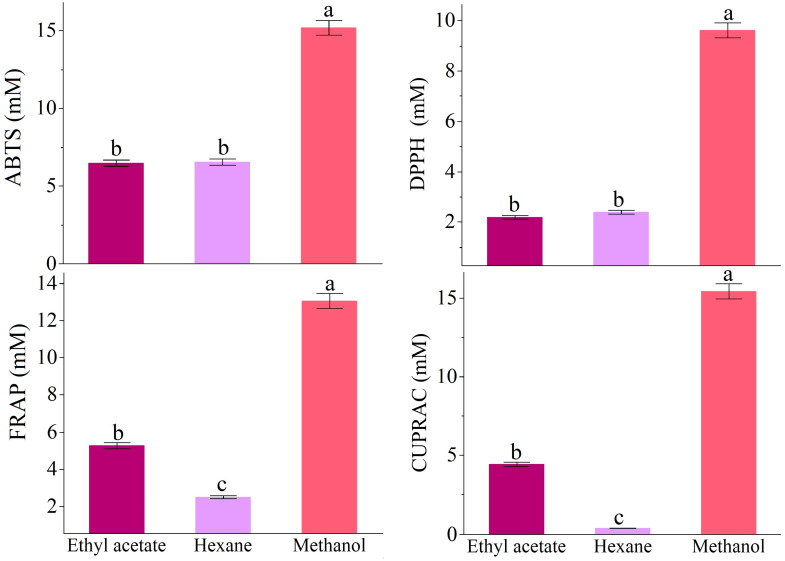
Fluctuations in antioxidant activities of RC influenced by the solvents. Different letters on the top of the bars indicate significant differences in *p* < 0.05 according to Fisher’s LSD test.

The total antioxidant capacities of the RC extracts were assessed using various methods. The ABTS value for the RC extracts was 15.20 mM for methanol, which was significantly higher than the 6.47 mM for ethyl acetate and the 6.54 mM for hexane. In the DPPH assay, the methanol extract showed the highest value at 9.62 mM, while ethyl acetate and hexane had lower values of 2.18 mM and 2.39 mM, respectively. For the FRAP method, the methanolic extract again performed best with a value of 13.07 mM, compared to 5.27 mM for ethyl acetate and 2.49 mM for hexane. The CUPRAC method revealed the methanol extract’s highest capacity at 15.44 mM, whereas ethyl acetate had a value of 4.43 mM and hexane was lower at 0.38 mM (Figure 2).

The research by Aghajanyan et al. [33] highlights that the seeds of *Rumex obtusifolius* contain significant levels of total phenolic compounds, with the highest concentrations found in ethanol extracts. These phenolic compounds are renowned for their antioxidant properties, contributing to the therapeutic effects of the plant. Similarly, a study by Çoruh et al. [34] reports that *R. crispus* has a TPC of approximately 56.31 µg/mg dry weight, further underscoring the high phenolic content of *Rumex* species.

Flavonoids, another crucial group of phytochemicals, show considerable variations across different *Rumex* species. The research by Sganzerla et al. [35] indicates that *R. obtusifolius* has a substantial flavonoid content, which may enhance its antioxidant activity. The concentration of flavonoids is important not only for the antioxidant potential but also for various health benefits, such as anti-inflammatory and anticancer properties.

Anthocyanins, a subclass of flavonoids, have also been identified in *Rumex* species. Ereifej et al. [36] reported a concentration of anthocyanins in *Rumex* acetosella at approximately 100.1 mg per 100 g. The presence of these compounds is significant because of their contribution to coloration and potential health benefits, including cardiovascular protection. Although less studied, protein and carbohydrate contents are essential for assessing the nutritional aspects of *Rumex* species. While specific data on the total protein and total carbohydrates in *Rumex* species are limited, it is established that many wild plants, including those from the Rumex genus, can provide essential nutrients. Gıdík’s [37] study on the *Berberis* species suggests that wild plants serve as significant sources of both bioactive compounds and essential nutrients, indicating a similar potential for *Rumex* species.

*Rumex* species are potent sources of bioactive compounds, particularly the total phenolics, flavonoids, and anthocyanins, which provide a substantial antioxidant activity. While the information on protein and carbohydrate levels in Rumex species is not extensively reported, the existing data highlights their nutritional and therapeutic potential, warranting further investigation.

### 3.3. Cytotoxicity Results

The ANOVA results demonstrated that the method of extraction, the concentration, the exposure duration, and their two-way and three-way interactions all had a statistically significant effect on cell viability (*p* ≤ 0.0001). As shown in Table 1, the exposure time had the most pronounced effect on cell viability (F = 6247.31 ***), followed by the solvent × time interaction (F = 459.77 ***), the solvent × time × concentration interaction (F = 49.08 ***), the concentration (F = 115.46 ***), the solvent × concentration interaction (F = 28.14 ***), the solvent (F = 21.95 ***), and the time × concentration interaction (F = 9.98 ***) (Table 1).

**Figure 3:**
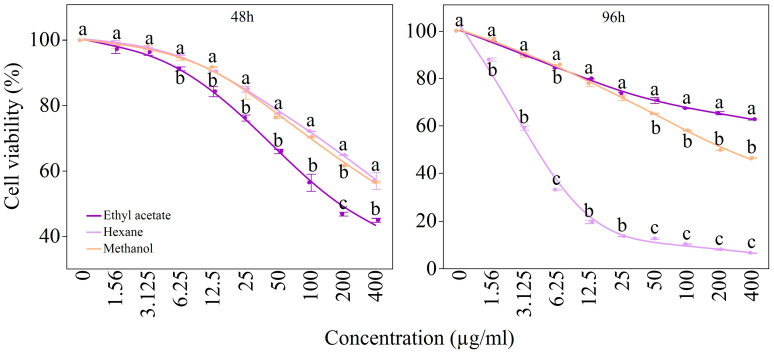
The impact of RC extracted by different solvents on cell viability at 48 and 96 h (Mean ± std. dev) compared to the control. Different letters on top of the points indicate significant differences according to Fischer’s LSD with an α of 0.05.

The results indicated that the cell viability in MCF-7 breast cancer cells decreased as the concentration of three different solvents increased, showing an inverse relationship. Additionally, cell death increased with longer treatment durations of 48 and 96 h for the cancerous breast cells. Cells exposed to the treatment for 48 h showed a decrease in viability with increasing concentrations from all solvents. Similarly, breast cancer cells treated for 96 h also demonstrated significant effects with higher concentrations. The ethyl acetate extract was more effective when MCF-7 breast cancer cells were exposed for 48 h, while the hexane extract exhibited a stronger effect on cell death at 96 h. The effectiveness of the hexane application significantly increased at the 96 h mark, with the mortality rate of MCF-7 cells increasing alongside higher doses. After 96 h of exposure, the cell viability dropped to 6.55% at a concentration of 400 µg/mL. Notably, even a low concentration of 12.5 µg/mL of the hexane extract resulted in over 80% cell death in MCF-7 cells (Figure 3). An increased production of reactive oxygen species (ROS) in cells can lead to toxic effects and cellular apoptosis, as well as promote lipid peroxidation, as a result of the presence of anthraquinones [38]. Studies have shown that anthraquinones can be found in herbal extracts and that these compounds can affect cancer cells via oxidative stress [39]. In addition, it is thought that harmful compounds involved in lipid peroxidation not only kill cells but also may be an important factor in cancer development [40]. HNE (4-hidroksi-2-nonenal) and similar lipid peroxidation products are directly related to increased oxidative stress in cells. This can affect cell functions and trigger apoptosis (cell death) processes [41,42].

A study on *R. acetosella* reported significant cytotoxic effects on MCF-7 cells, indicating that these extracts inhibited cell proliferation in a dose-dependent manner, suggesting potential applications of *Rumex* spp. in breast cancer treatment [43]. Another study on the *Rumex dentatus* plant extract demonstrated the ability of this species to inhibit cell proliferation, arrest the cell cycle, and induce apoptosis in triple-negative breast cancer cell line MDA-MB-231 cells. Batool et al. [44] used an MTT assay for a cytotoxicity assessment and demonstrated its efficacy against aggressive breast cancer cells through the suppression of the NF-κB pathway, a known regulator of cancer cell survival. In another recent study, dried root extracts of *R. abyssinicus* were tested for cytotoxic effects on MCF-7 breast cancer cells, and researchers reported that this extract also showed significant cytotoxic effects [11]. A similar result was obtained in *R. crispus*, exhibiting a potential cytotoxic activity against MCF-7 cells [45]. Nasr et al. [46] reported a moderate anticancer activity against breast cancer cells treated by the *Rumex vesicarius* extract and suggested it as a potential therapeutic agent in treatment regimens. Abou Elfotoh et al. investigated several *Rumex* species and found that some showed a notable cytotoxic activity against MCF-7 and other cancer cell lines, highlighting the need for further targeted studies [47]. Berillo et al. [8] and Li et al. [6] reported that extracts from various *Rumex* species exhibit antibacterial, antitumor, and anti-inflammatory properties. The findings of this study further support their health-promoting effects, particularly against MCF-7 human breast cancer cells.

In this study, we evaluated RC extracts prepared with different solvents and, for the first time, demonstrated their significant cytotoxic effects on MCF-7 breast cancer cell lines, in line with findings on other *Rumex* species in the literature.

### 3.4. IC_50_ Determination

After 48 h, the IC_50_ value ranged from 29.74 ± 5.58 µg/mL for the ethyl acetate extract (RC-ethyl acetate) to 46.22 ± 6.85 µg/mL for the hexane extract (RC-hexane). However, after 96 h of treatment, the values changed, ranging from 3.93 ± 0.03 µg/mL for the hexane extract (RC-hexane) to 20.61 ± 5.5 µg/mL for the methanol extract (RC-methanol). The RC-hexane extract demonstrated a pronounced increase in cytotoxicity over time, with its IC_50_ decreasing from 46.22 ± 6.85 µg/mL at 48 h to 3.93 ± 0.03 µg/mL at 96 h. The IC_50_ values of the ethyl acetate and methanol extracts also decreased noticeably over 96 h, while the cell proliferation exceeded the cell death at this time point (Table 2). Doxorubicin displayed an IC_50_ of approximately 0.68 to 0.75 µg/mL in parental MCF-7 cells, but this value rises to 5.78 µg/mL in doxorubicin-resistant derivatives (MCF-7/Dox) [48,49,50]. This notable increase underscores the adaptive nature of MCF-7 cells in response to chemotherapeutic agents, illustrating the complexity of cancer drug resistance. Similar trends are observed with tamoxifen, where IC_50_ values typically range from 0.1 to 1 μM, but resistant variants, such as MCF-7/TAM-R, reflect a marked insensitivity with values reaching up to 14 μM [51,52,53]. In contrast, paclitaxel exhibits a broader IC50 range from 1 to 30 nM, which is significantly influenced by drug formulation, with a recent study reporting an IC50 of 1.5 nM for free paclitaxel [54], while a nanoparticle formulation led to a considerably higher IC50 of 175 µg/mL [55]. Notably, recent investigations into *Phyllanthus niruri* fractions revealed that Fr-3 displayed a considerable cytotoxicity against both MCF-7 and MCF-7ADR cells, with IC_50_ values of 97.2 ± 1.13 μg/mL and 51.1 ± 0.93 μg/mL, respectively. Bal et al. [56] reported an IC_50_ value of 865.4 μg mL^−1^ for MCF-7 treated with calcium alginate-encapsulated *Olea europea* leaf extracts. Although standard drug chemicals show slightly better effects than RC-hexane, the significantly high cytotoxicity compared to other plant extracts suggests the anticancer potential of RC against human breast cancer cell lines, such as MCF-7.

### 3.5. Real-Time qPCR Analysis

**Figure 4:**
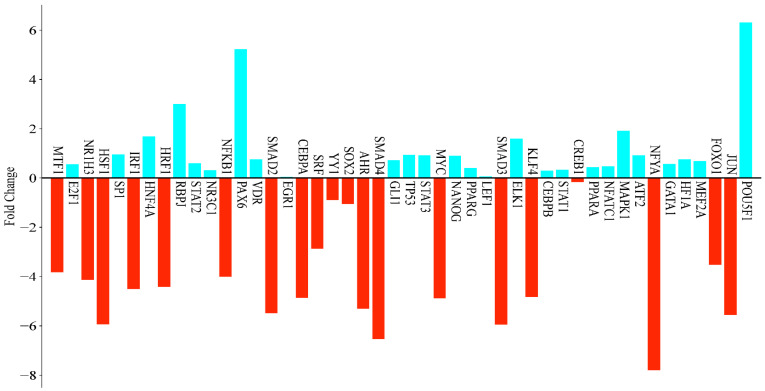
Gene expression levels of RC-hexane extracts in human breast cancer cell MCF-7.

In this study, the gene expression of hexane extracts from RC was analyzed using 46 genes. The results showed that 20 genes were downregulated while 26 genes were upregulated in response to the hexane extract. Notably, six of the upregulated genes exhibited increases of threefold or more, indicating significant differences. The gene with the highest level of upregulation was *POU5F1*, which increased by 79.34-fold, followed by *PAX6* at 37.27-fold and *RBPJ* at 7.94-fold. Among the downregulated genes, *NFYA* experienced the most substantial decrease at −222.86-fold, followed by *SMAD4* at −93.05-fold, *SMAD3* at −61.82-fold, and *HSF1* at −61.39-fold (Figure 4).

In this study, the treatment of MCF-7 breast cancer cells with the RC extract resulted in significant changes in gene expression, with several transcription factors (TFs) exhibiting more than a twofold increase in their expression levels.

Among the upregulated TFs, *POU5F1* (*OCT4*) showed the most substantial increase, with a fold change of +79.34, indicating a strong activation of transcriptional programs associated with pluripotency. Additionally, *PAX6* (+37.27), which is critical for the commitment to the neuroectodermal lineage, and *RBPJ* (+7.94), an important component of the Notch signaling pathway, were also significantly upregulated.

Moderate increases were also observed in *MAPK1* (+3.78), *HNF4A* (+3.23), *ELK1* (+3.03), *SP1* (+1.93), *TP53* (+1.92), *STAT3* (+1.88), *ATF2* (+1.88), *NANOG* (+1.85), *GLI1* (+1.65), *VDR* (+1.68), HF1A (+1.68), *GATA1* (+1.48), *E2F1* (+1.46), *PPARA* (+1.36), *PPARG* (+1.32), *STAT2* (+1.51), *STAT1* (+1.26), *CEBPB* (+1.22), *NR3C1* (+1.24), *NFATC1* (+1.39), and *EGR1* (+1.04).

The upregulation of certain genes indicates the activation of signaling networks that are associated with cell survival, metabolism, nuclear receptor signaling, and transcriptional regulation. Conversely, there were significantly more downregulated transcription factors, which exhibited greater fold changes. *NFYA* demonstrated the most considerable suppression, with a fold change of −222.86. This suggests a potential global repression of the core promoter activity and general transcriptional machinery. Several components of the TGF-β signaling pathway were also significantly downregulated, including SMAD4 (−93.05), *SMAD3* (−61.82), and *SMAD2* (−44.63), indicating a strong inhibition of tumor-suppressive TGF-β responses. Additionally, the stress response and heat shock regulator *HSF1* showed a notable decrease (−61.39), as did *JUN* (−46.85), which is a key component of the AP-1 complex involved in cell proliferation and survival. AHR (−39.40), a xenobiotic sensor that plays a role in detoxification and immune regulation, was also significantly downregulated.

The upregulation of pluripotency-related genes (*POU5F1* (*OCT4*) and *NANOG*) in MCF-7 cells treated with the RC hexane extract is remarkable for the acquisition of cancer stem cell-like properties. Clinically, the acquisition of a stem cell-like phenotype has often been associated with tumor plasticity, a resistance to treatment, metastasis, and a risk of recurrence [57]. However, in our study, the upregulation of pluripotency genes was observed simultaneously with an increased apoptosis activity. Hoechst 33,258 staining and a significant decrease in cell viability support this. This suggests that the early activation of stem cell-like pathways may be a transient stress-induced response that occurs before apoptosis. Similar paradoxes have also been reported in the literature. In some cases, cancer cells may temporarily increase stem cell markers when exposed to stress, which is usually a type of stress response rather than a permanent acquisition of stem cell properties [58].

Additional significantly downregulated TFs included *CEBPA* (−29.04), *MYC* (−29.45), *KLF4* (−28.44), *IRF1* (−22.63), *NRF1* (−21.41), *NR1H3* (−17.51), *NFKB1* (−16.00), *MTF1* (−14.12), and *FOXO1* (−11.47), many of which are involved in the immune response, metabolic regulation, differentiation, and stress adaptation. The downregulation of SRF (−7.31), a factor involved in cytoskeletal dynamics and immediate-early gene expression, further supports the idea of reorganization in both the cytoskeletal structure and transcriptional activity. Collectively, these changes in gene expression suggest that RC may influence transcriptional programs in MCF-7 cells, including those associated with the stemness, cell cycle progression, stress response, differentiation, and immune regulation.

### 3.6. Metabolic Responses of Cancer Cells/Signaling Pathways

In our analysis of the expression values of 46 genes from the Reactome database, we found that the application of RC-hexane extracts significantly influenced several metabolic pathways in the MCF-7 cancer cell line. This analysis provided insights into the effects of the RC extract on MCF-7 breast cancer cells. A key finding was the enrichment of the Nuclear Receptor Transcription Pathway, which involves transcription factors such as *HNF4A*, *VDR*, *PPARG*, *PPARA*, and *NR3C1*. These factors are crucial for regulating metabolic, hormonal, and inflammatory responses, suggesting that the extract may impact the cellular metabolism and growth.

The extract also enriches the Transcriptional Regulation of Multipotent Stem Cells pathway, leading to the upregulation of the key regulators *POU5F1* (*OCT4*), *NANOG*, and *SOX2*, while downregulating differentiation markers like *SMAD2*, *SMAD4*, and KLF4. This suggests that the extract may modulate the expression of genes involved in cellular metabolism and growth. Furthermore, the analysis reveals a substantial enrichment in the Generic Transcription Pathway and RNA Polymerase II Transcription, supporting the idea of a broad transcriptional reset. Changes in developmental pathways such as Gastrulation and Germ Layer Formation, influenced by transcription factors like *SMAD2*/*3*/*4* and *FOXO1*, suggest a disturbance in differentiation processes. Alterations in Cytokine Signaling and MAP Kinase Activation suggest modified immune responses, with the downregulation of factors like *IRF1* and *JUN* and the upregulation of *STAT3* and RBPJ, which may indicate immune evasion mechanisms.

The pathway analysis results derived from the Reactome platform provide compelling insights into the molecular impact of the RC extract on MCF-7 breast cancer cells. The most prominent finding is the significant enrichment of the Nuclear Receptor Transcription Pathway, underscoring the involvement of ligand-dependent transcription factors such as *HNF4A*, *VDR*, *PPARG*, *PPARA*, and *NR3C1*. These factors play critical roles in modulating metabolic, hormonal, and inflammatory responses, and their differential expression suggests that the extract may influence cellular metabolism and growth through hormonal regulation.

**Figure 5:**
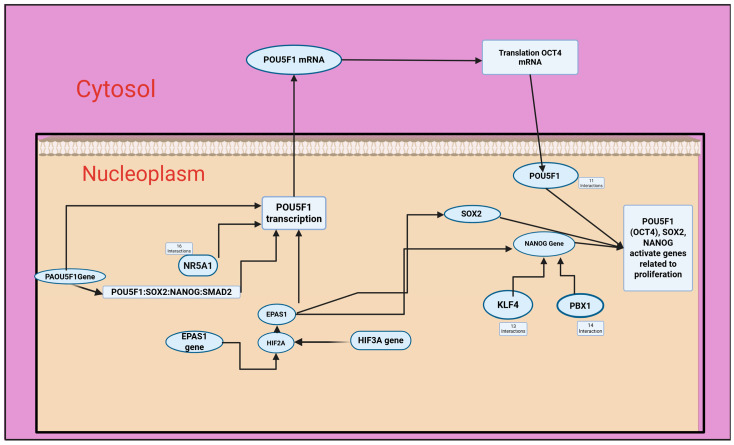
Transcriptional regulation pluripotent stem cells in MCF-7 cancer cell line by RC-hexane extract. Created in BioRender. TUTAR, Y. (2025) https://BioRender.com/kvxg7ol, accessed on 29 May 2025.

Another key observation is the upregulation of the core pluripotency regulators *POU5F1* (*OCT4*), *NANOG*, and *SOX2*, alongside the enrichment of the Transcriptional Regulation of Pluripotent Stem Cells pathway. These transcription factors are typically associated with embryonic stem cell maintenance and self-renewal. Their induction, coupled with the downregulation of differentiation markers such as *SMAD2*, *SMAD4*, and *KLF4*, suggests the modulation of pathways related to cell differentiation (Figure 5).

Inducing a stem cell-like state in cancer cells is clinically significant because it can lead to increased malignancy and a higher likelihood of tumor recurrence after treatment. Cancer stem cells (CSCs) possess the ability to self-renew and differentiate, and they are closely linked to treatment resistance and a poor patient prognosis [59,60,61]. This stem-like state is often associated with an epithelial–mesenchymal transition (EMT); through an EMT, differentiated cells can transition to a more invasive and resistant phenotype [62,63]. The *SLUG*/*STAT3* pathway, for example, plays a key role in regulating stem-like states, suggesting that targeting this pathway during treatment could prevent the development of more aggressive phenotypes [64,65].

The tumor microenvironment also plays a decisive role in inducing a stem cell-like phenotype. Growth factors and cytokines, such as TGF-β, contribute to stem cell differentiation by supporting cellular reprogramming [66,67]. In certain tumors, such as *MPNST*, elevated levels of markers such as *ALDH1A1* and *ZEB1* are associated with poor clinical outcomes [62,68]. In lung adenocarcinoma, hyaluronan receptors have been shown to enhance stem cell properties and chemotherapy resistance [69,70]. Furthermore, epigenetic modifications, such as the loss of histone *H2B* ubiquitination, have been associated with poorly differentiated, aggressive tumor phenotypes [68,71]. Together, these findings suggest that targeting CSCs could improve the treatment efficacy and overcome drug resistance [61,63,72,73].

**Figure 6:**
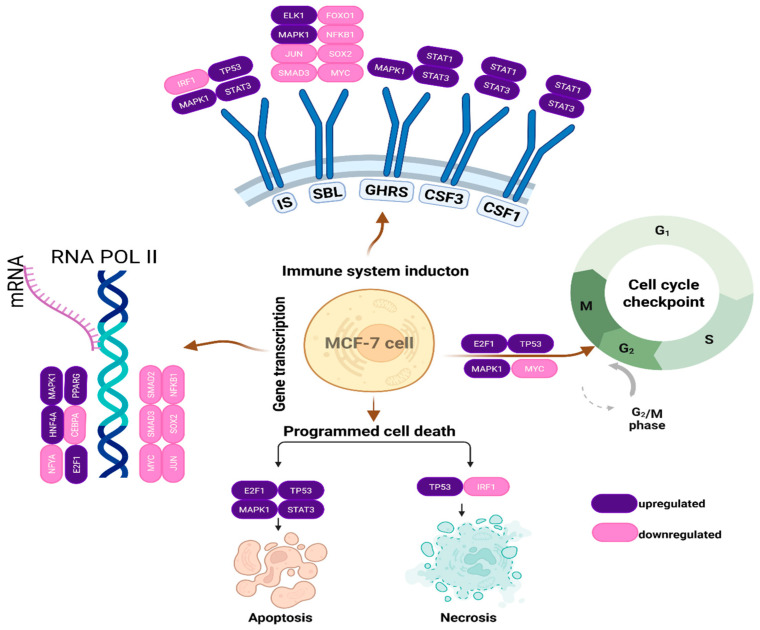
Metabolic pathways significantly influenced by the RC-hexane extract treatment, based on the expression of 46 genes. Created in BioRender. TUTAR, Y. (2025) https://BioRender.com/2gyuus0, accessed on 29 May 2025.

The broad activation of the transcriptional machinery, reflected by the enrichment in both the Generic Transcription Pathway and RNA Polymerase II Transcription, suggests the modulation of multiple transcriptional pathways. This is further supported by the modulation in transcription factors including *MYC*, *JUN*, *NFYA*, and *CREB1*, which were among the most significantly downregulated. Notably, *MYC* is a key driver of the cell cycle progression at both G1/S and G2/M checkpoints but also primes cells for apoptosis under stress conditions. *JUN* (*c-JUN*), depending on its binding partners, can either promote proliferation or apoptosis, particularly in response to stress signals. *NFYA* regulates genes essential for the G2/M transition, such as *Cyclin B1* and *CDK1*, and its repression could contribute to cell cycle arrest. *CREB1*, which typically exerts anti-apoptotic effects through the regulation of *BCL-2* family genes and facilitates mitotic entry via *CDC25B* and *Cyclin A*/*B*, is similarly downregulated, potentially enhancing apoptotic susceptibility and impairing mitotic progression (Figure 6).

Significant changes were also observed in pathways related to Developmental Biology, particularly Gastrulation, Germ Layer Formation, and Adipogenesis, which are governed by transcription factors like *SMAD2*/*3*/*4*, *FOXO1*, and *CEBPA*. These pathways suggest the extract’s influence on developmental gene regulatory networks, potentially disrupting the cancer cell differentiation and lineage commitment.

Furthermore, the identification of the altered activity in the Cytokine Signaling, MAP Kinase Activation, and Transcriptional Regulation of Granulopoiesis points to the modulation of immune and inflammatory responses. The downregulation of immune-related transcription factors such as *IRF1*, *JUN*, and *CEBPA*, alongside the upregulation of factors such as *STAT3* and *RBPJ*, may indicate a shift in the immune regulatory environment of the cells, possibly contributing to immune evasion or altered tumor–immune interactions.

From a statistical perspective, the most significantly enriched pathways include the Nuclear Receptor Transcription Pathway (FDR: 1.27 × 10^−14^), Generic Transcription Pathway (FDR: 5.25 × 10^−14^), RNA Polymerase II Transcription (FDR: 3.29 × 10^−13^), and Transcriptional Regulation of Pluripotent Stem Cells (FDR: 3.52 × 10^−13^). The very low false discovery rates observed across these pathways strongly support the robustness and reliability of enrichment results. Additionally, pathways related to Developmental Biology, MAPK signaling, and Immune System Modulation also showed high statistical significance, further confirming that the RC treatment alters fundamental cellular programs including transcriptional control, developmental plasticity, and immunological signaling.

Taken together, these findings suggest that the RC extract modulates multiple transcriptional and signaling pathways in MCF-7 cells, including those related to pluripotency, differentiation, and immune signaling. Further functional studies and validation in vivo models are warranted to delineate the therapeutic relevance of these molecular changes.

### 3.7. Flow Cytometry Analyses

#### 3.7.1. Cell Cycle

**Figure 7:**
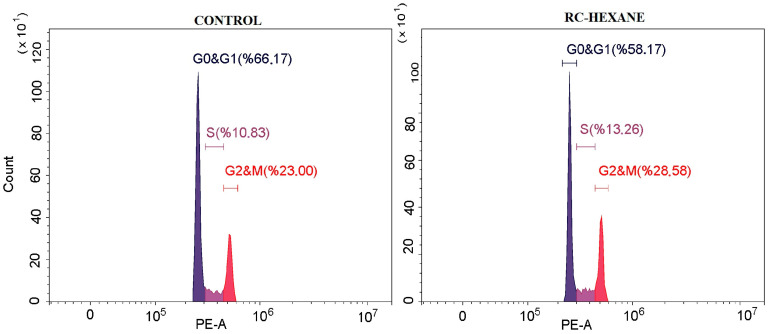
The cell cycle analysis of MCF-7 cancer cells under control and RC-hexane treatments.

The effects of the RC-hexane extract on the cell cycle of MCF-7 breast cancer cells were evaluated (Figure 7). The findings indicated an increase in both the G2/M and S phases, while the G0/G1 phase exhibited a lower rate compared to the control group. Specifically, the extract resulted in a decrease in the G0/G1 phase from 66.17% to 58.17%, along with a significant increase in the S phase from 10.83% to 13.26% and in the G2/M phase from 23.00% to 28.56%. These results suggest that the RC-hexane extract induced an arrest in the G2/M and S checkpoints of MCF-7 cancer cells (Supplementary Table S1).

The research indicates that RC plant extracts can induce a cell cycle arrest in MCF-7 breast cancer cells, particularly at the G2/M phase. However, some extracts primarily affect the G0/G1 phase. One specific compound, BHMC—derived from certain plant sources—has been shown to induce cell cycle arrest at the G2/M phase in MCF-7 cells, ultimately leading to apoptosis after treatment [74]. This finding highlights the significant impact of compounds targeting the G2/M checkpoint, which may facilitate apoptosis and offer potential therapeutic options for breast cancer treatments.

Studies involving extracts from *Neolamarckia cadamba* indicate that these extracts primarily cause cell cycle arrest in the G0/G1 phase [75]. These findings highlight the various mechanisms by which different plant extracts exert their anticancer effects on MCF-7 cells. Salidroside from Rhodiola rosea has been shown to induce cell cycle arrest at the G0/G1 phase as well [76,77]. Additionally, curcumin has demonstrated the ability to induce a G0/G1 arrest under standard conditions; however, in certain cancer cell variations, it can lead to a G2/M arrest, suggesting that its effectiveness may depend on concentrations [78]. Further investigations into plant-derived compounds reveal that various bioactive substances can effectively induce a G2/M phase arrest. For example, Withaferin A has been found to cause cell cycle arrest specifically at the G2 and M phases in MCF-7 cells, regardless of the p53 status. This finding is significant since p53 mutations are common in many cancer types, including breast cancer [79].

Interestingly, additional studies have shown that apoptosis and cell cycle arrest are often interconnected outcomes of the exposure to polyphenolic compounds. Extracts rich in polyphenols have demonstrated their ability to induce cell cycle arrest while simultaneously promoting the generation of ROS. This generation of ROS has been linked to both cell cycle regulation and the induction of apoptosis [80]. These findings suggest that plant-based compounds may enhance their efficacy in cancer treatment by modulating cell cycle checkpoints.

The cell cycle of MCF-7 breast cancer cells is significantly affected by various compounds, including natural extracts and synthetic derivatives, which can lead to cell cycle arrest and apoptosis. Research has shown that certain extracts from plants, particularly *Rumex vesicarius*, display a selective cytotoxicity against MCF-7 cells, effectively promoting cell death at higher concentrations. A study indicated that methanol extracts resulted in growth inhibition in a dose-dependent manner, leading to a significant reduction in the population of viable cells after treatment [81].

In terms of cell cycle regulation, the Rumex extracts, along with other cancer therapeutics, have been documented to induce cell cycle arrest at specific phases. Li et al. demonstrated that the Si1 gene can cause cell cycle arrest at the G2/M phase in MCF-7 cells, linking this arrest to an increase in markers of apoptosis and autophagy [82]. Supporting this, Moradi-Gharibvand et al. [83] found that combining pomegranate seed extract with adipose-derived mesenchymal stem cells (ADSCs) resulted in the G1 phase arrest in MCF-7 cells, highlighting the diverse mechanisms through which compounds can influence cell cycle phases, ultimately leading to tumor cell reduction.

Furthermore, several studies have investigated the impact of various treatments on MCF-7 cell cycle dynamics. Taheri et al. [84] demonstrated that different durations of treatment with specific consciousness fields could affect cell viability and induce changes in apoptotic pathways within these cells. Additionally, Zhou et al. [85] provided insights into how compounds like curcumin can enhance the efficacy of other chemotherapeutics, suggesting that the regulation of checkpoints such as G2/M and the involvement of proteins like cyclin-dependent kinases are central to these processes.

Interestingly, certain pathways, including *MAPK* and *PI3K* signaling, have been shown to influence cell cycle arrest and the induction of apoptosis, further linking the biochemical responses triggered by these treatments [86]. This complex interplay underscores the potential of therapeutic agents from natural sources to not only inhibit tumor growth but also to regulate critical mechanisms governing cancer cell dynamics. In addition to promoting G0/G1 phase arrest, plant extracts can also induce G2/M phase arrest. Kuo et al. [87] demonstrated that 2-methoxyestradiol (2-MeO-E2) caused cell cycle arrest at the G2/M phase in MCF-7 cells, reducing the number of cells in the G1/G0 phase.

Our findings indicated that there was an arrest in the G2/M phase, which supports earlier reports regarding the effects of plant-derived extracts on MCF-7 cancer cells. This research suggests that plant extracts, including RC, may have potential for a further investigation as candidates for breast cancer treatment.

In summary, the evidence indicates a variety of effects from plant extracts on MCF-7 breast cancer cells, with some compounds primarily affecting the G0/G1 phase and others distinctly influencing the G2/M phase. This variability suggests that the therapeutic application of these extracts could be tailored according to their specific actions on the cell cycle, providing a promising avenue for the development of more effective cancer therapies.

#### 3.7.2. Apoptosis

**Figure 8:**
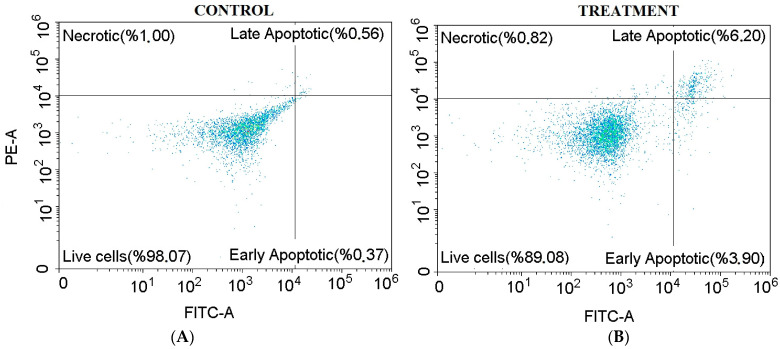
Apoptosis in MCF-7 cancer cells by the treatment of the control (**A**) and RC-hexane (**B**).

The effects of RC-hexane on apoptosis in MCF-7 cancer cells were analyzed through flow cytometry, as shown in Figure 8. After a 96 h treatment, the live cell rate decreased from 98.07% in the control group to 89.08%. This decrease included 3.90% of cells undergoing early apoptosis, 6.20% in late apoptosis, and 0.82% as necrosis. In the control group, these rates were recorded as 0.37%, 0.56%, and 1.00%, respectively (Supplementary Table S2). The MCF-7 cell line, derived from human breast cancer, is a vital model for studying the effects of various treatments on apoptosis or programmed cell death.

The role of flavonoids in inducing apoptosis has been documented. Kaempferol has been reported to inhibit cell proliferation and induce apoptosis in pancreatic cancer cells via the FAS pathway, suggesting its potential utility in cancer prevention and therapy [88]. Similarly, naringin was found to downregulate the PI3K/Akt/mTOR signaling cascade, leading to autophagy-mediated growth inhibition in gastric cancer cells [89]. This evidence aligns with findings indicating that flavonoids can modulate factors associated with apoptosis [90].

Moreover, the antioxidant capabilities of flavonoids contribute significantly to their anticancer effects. The generation of ROS is a common mechanism through which flavonoids exert cytotoxic effects on cancer cells while sparing normal cells. The flavonoid morin has been associated with its ability to induce apoptosis by modulating Bcl-2 family members and activating the Fas receptor in human colorectal cancer cells [91].

Clinical relevance is further supported by epidemiological studies that have highlighted the association between the dietary flavonoid intake and a reduced cancer risk. The intake of flavonoid-rich foods has been linked to lower incidence rates of breast, prostate, and colorectal cancers [92]. Reviews have synthesized findings across various studies, revealing that flavonoids not only inhibit carcinogenesis but may also reshape the gut microbiota to produce metabolites that exert protective effects against cancer [93].

The structural diversity of flavonoids contributes to their unique biological activities. Hesperetin has been reported to arrest the cell cycle at the G1 phase in breast cancer cells [94]. Additionally, flavonoids like quercetin exhibit antiproliferative effects through their action on estrogen receptors, while apigenin downregulates the androgen receptor expression in prostate cancer, further emphasizing their potential as targeted therapeutic agents [61].

In summary, plant extracts, such as RC-hexane extracts, can induce apoptosis in MCF-7 cells via various mechanisms. These include the modulation of apoptotic proteins, the generation of oxidative stress and mitochondrial dysfunction, the activation of caspases, and the presentation of distinct morphological changes. Additionally, the potential for synergistic effects with traditional therapies underscores the promise of these natural compounds in cancer treatment.

Research into the use of RC extracts alongside standard chemotherapeutic agents, such as tamoxifen, doxorubicin, and paclitaxel, shows promise in terms of increasing treatment efficacy. Previous studies suggest that this potential synergy may be due to the presence of phytochemicals with cytotoxic, antioxidant, and anti-inflammatory properties, such as anthraquinones and flavonoids, in the RC plant [8,95]. Anthraquinones in RC were reported to affect cellular pathways related to apoptosis and cell proliferation. This suggests that these components may enhance the efficacy of chemotherapeutic agents that induce apoptosis, such as doxorubicin and paclitaxel [95]. Furthermore, combining RC extracts with tamoxifen may enhance the antiproliferative activity, particularly in estrogen receptor-positive breast cancer cells. It has been hypothesized that phytochemicals derived from Rumex may modulate estrogen signaling pathways, thereby enhancing the cytotoxic effect of tamoxifen [8]. Agents such as doxorubicin and paclitaxel, which also affect the cell cycle and pathways related to apoptosis, may produce more effective clinical outcomes when used in combination with RC [20,96]. Furthermore, certain RC components have demonstrated the ability to inhibit the ABCB1 transporter, which is involved in multidrug resistance. This inhibition could contribute to overcoming drug resistance and enhancing the accumulation of chemotherapeutic agents within cells, thereby increasing their cytotoxic activity [97]. Furthermore, the anti-inflammatory properties of RC may reduce tumor-associated inflammation, enhancing treatment efficacy [96].

## 4. Conclusions

The extraction efficiency of bioactive compounds from RC varies significantly depending on the choice of solvent. Methanol, ethyl acetate, and hexane demonstrated optimal results for the antioxidant activity, anthocyanin content, and carbohydrate extraction, respectively. The RC extract induced apoptosis, mostly late apoptosis. The cell cycle analysis showed that the RC extract arrests the cycle in S and G2-M checkpoints. Previous studies have highlighted the promising cytotoxic effects of Rumex species on MCF-7 breast cancer cells, indicating their potential for developing anticancer treatments, and our transcriptomic analysis further reveals the modulatory impact of the RC extract on several regulatory pathways, including nuclear receptor signaling and immune responses, which may contribute to its anticancer properties. Notably, there is an upregulation of pluripotency-associated genes alongside a downregulation of genes involved in differentiation and apoptosis. Additional research is needed to explore the therapeutic implications and mechanisms of action related to these findings. These preliminary findings suggest that the combination of RC extracts and chemotherapeutic agents should be evaluated in clinical settings.

Further research into dosage protocols, pharmacokinetics, and target cancer types for combination therapy will be crucial in translating this synergy into clinical practice. The validation of these interactions through randomized controlled trials, in particular, will contribute to the development of more effective cancer treatment formulations.

## Figures and Tables

**Table 1:** Results of 3-way ANOVA on cell viability.

Variation Source	DF	Sum of Squares	F Ratio	*p*-Value
Solvent	2	471.71	21.95	7.55 × 10^−9^
Time	1	67,143.11	6247.31	2.4 × 10^−105^
Concentration	9	2481.76	115.46	1.48 × 10^−88^
Solvent × Time	2	44,472.31	459.77	1.09 × 10^−28^
Solvent × Concentration	18	5444.79	28.14	1.19 × 10^−34^
Time × Concentration	9	965.34	9.98	2.5 × 10^−11^
Solvent × Time × Concentration	18	9495.71	49.08	1.24 × 10^−46^

DF: degree of freedom.

**Table 2:** IC_50_ values (µg/mL) of extracts from RC.

Source	48 h	96 h
Hexane	46.22 ± 6.85 a	3.93 ± 0.03 c
Ethyl acetate	29.74 ± 5.58 b	10.43 ± 2.74 b
Methanol	42.19 ± 8.4 a	20.61 ± 5.5 a

Different letters in the same columns indicate significant differences at *p* ≤ 0.05 according to Fisher’s LSD test.

## Data Availability

The data that support the findings of this study are available on request from the corresponding author.

## Supplementary Materials

The following supporting information can be downloaded at https://www.mdpi.com/article/10.3390/antiox14070879/s1. Table S1: The cell cycle analysis in MCF-7 human breast cancer cells. Table S2: The apoptosis analysis in MCF-7 human breast cancer cells.

## Abbreviations

The following abbreviations are used in this manuscript:

RC	*Rumex confertus*
FRAP	Ferric reducing antioxidant power
CUPRAC	Cupric reducing antioxidant capacity
ABTS	2,2′-azino-bis(3ethylbenzothiazoline-6-sulfonic acid
DPPH	2,2-diphenyl-1-picrylhydrazyl
TPC	Total phenolic content
TFC	Total flavonoid content
Ant	Total anthocyanin content
Carb	Total soluble carbohydrates
Prot	Total soluble proteins
MTT	[3-(4,5-dimethylthiazol-2-yl)-2,5-diphenyltetrazolium bromide]
PCR	Polymerase chain reaction
DMEM	Dulbecco’s modified eagle medium
EDTA	Ethylenediaminetetraacetic acid
DMSO	Dimethyl sulfoxide
