# Supplementary material for: In Vitro Effects of Rumex confertus Extracts on Cell Viability and Molecular Pathways in MCF-7 Breast Cancer Cells
Source: Antioxidants (Basel). 2025 Jul 18;14(7):879. doi: 10.3390/antiox14070879 (PMC12291928; doi:10.3390/antiox14070879)
Supplement: Supplementary file 1 [file antioxidants-14-00879-s001.zip › antioxidants-3707666-supplementary.pdf]

Supplementary Materials

Supp. Table 1. The cell cycle analysis in MCF-7 human breast cancer cells

Treatment	G0/G1	S	G2/M
Control	66.17 ± 3.31 a	10.83 ± 0.54 b	23.00 ± 1.15 b
Rumex confertus (Hexane extract)	58.17 ± 2.91 b	13.26 ± 0.66 a	28.56 ± 1.43 a

Different letters in the same column indicate significant differences at $p \leq 0.05$ according to Student's t test.

Supp. Table 2. The apoptosis analysis in MCF-7 human breast cancer cells

Treatment	Live cell	Early Apoptotic	Late Apoptotic	Necrotic
Control	98.07 ± 4.90 a	0.37 ± 0.02 b	0.56 ± 0.03 b	1.00 ± 0.05 a
R. confertus (Hexane extract)	89.08 ± 4.45 a	3.90 ± 0.20 a	6.20 ± 0.31 a	0.82 ± 0.04 b

Different letters in the same column indicate significant differences at $p \leq 0.05$ according to Student's t test.
